# Testing students’ e-learning via Facebook through Bayesian structural equation modeling

**DOI:** 10.1371/journal.pone.0182311

**Published:** 2017-09-08

**Authors:** Hashem Salarzadeh Jenatabadi, Sedigheh Moghavvemi, Che Wan Jasimah Bt Wan Mohamed Radzi, Parastoo Babashamsi, Mohammad Arashi

**Affiliations:** 1 Department of Science and Technology Studies, University of Malaya, Kuala Lumpur, Malaysia; 2 Department of Operation and Management Information System, University of Malaya, Kuala Lumpur, Malaysia; 3 Department of Language Education and Humanities, University Putra Malaysia, Serdang, Malaysia; 4 Department of Applied Mathematics, Shahrood University of Technology, Shahrood, Iran; Southwest University, CHINA

## Abstract

Learning is an intentional activity, with several factors affecting students’ intention to use new learning technology. Researchers have investigated technology acceptance in different contexts by developing various theories/models and testing them by a number of means. Although most theories/models developed have been examined through regression or structural equation modeling, Bayesian analysis offers more accurate data analysis results. To address this gap, the unified theory of acceptance and technology use in the context of e-learning via Facebook are re-examined in this study using Bayesian analysis. The data (S1 Data) were collected from 170 students enrolled in a business statistics course at University of Malaya, Malaysia, and tested with the maximum likelihood and Bayesian approaches. The difference between the two methods’ results indicates that performance expectancy and hedonic motivation are the strongest factors influencing the intention to use e-learning via Facebook. The Bayesian estimation model exhibited better data fit than the maximum likelihood estimator model. The results of the Bayesian and maximum likelihood estimator approaches are compared and the reasons for the result discrepancy are deliberated.

## Introduction

The use of information systems and the Internet as teaching tools is a noteworthy aspect in today's tech-savvy community. The reason is that these tools are expanding rapidly into education, while teaching without technology is now seen as uninteresting [1]. According to various researchers, e-learning is usually associated with face-to-face activities [2]. It is also a complementary tool to traditional learning and teaching processes, since e-learning can facilitate education and training through information communication technology (ICT) for anyone, anytime and anywhere. Some organizations utilize e-learning for employee training, as it lowers training costs, increases learning flexibility (place and time), and enables on-demand training [3]. In view of the advantages of e-learning, numerous universities and educational institutions offer new and innovative means of delivering education to students [4]. A number of academic institutions and universities apply blended pedagogical approaches, thus enabling instructors to use both traditional and digital domains [1]. Other universities use simulation games, reflective learning [1, 5] and films [6] as complementary tools to their existing teaching systems. E-learning is a rapidly spreading system that facilitates student participation in courses [7].

Among the recent, most frequently used technologies in business, marketing advertising and learning are social networks. Although social networks were created to foster personal relationships [8], many realize the effectiveness of utilizing this technology for other purposes, including business and teaching. Researchers argue that social media/social network use for teaching and learning is one of the main revolutions to have taken place in academia over the last few years [9]. Social network sites embrace collaborative learning and enhance communication by encouraging members to work in personalized environments [10, 11]. These sites can function as education tools because they enable communication between students and their instructors and facilitate class discussions and information distribution [12, 13].

Students have already adopted social network sites (SNSs) tremendously, and experience shows that SNSs are not only tools for connecting friends and family but are also a strong medium for parallel journalism and the mobilization of a new generation of students [14].

Considering the growing use of social networks, numerous researchers have investigated social network acceptance and use behavior in different contexts, such as teaching and learning [4, 9]. Various theories and models have been applied to measure individual intention to use this technology. The unified theory of acceptance and use of technology (UTAUT) was developed by Venkatesh, Morris, Davis, and Davis [15] in 2003 to identify the factors affecting intention to use new technology. Venkatesh, Morris, Davis, and Davis [15] conducted a longitudinal study and applied partial least squares regression to test employees’ acceptance of new technology. This model explained 77% of the variance in behavioral intention and 52% of the variance in use behavior. Venkatesh, Thong, and Xu [16] expanded UTAUT to the customer behavior context and proposed and tested UTAUT2, which incorporates new constructs including price value, hedonic motivation and habit. UTAUT2 explained 74% of the variance in behavioral intention to use a technology and 52% of the variance in consumers’ use behavior. Venkatesh and most other researchers who have employed the UTAUT and UTAUT2 models applied regression or structural equation modeling (SEM) to test their research models. Other researchers in the statistics field argue that some techniques are able to produce better results than others.

Various estimation procedures are available for SEM estimation, but the most common is the maximum likelihood (ML) estimator. Analyses using the ML estimator often suffer from model misspecification because the models are too strict with exact zero cross-loadings and zero residual correlations, which may cause poor model fit [17] and extensive parameter bias in factor loadings and factor correlations [18, 19]. Models imposing these strict criteria are often rejected [20], leading researchers to invoke a series of model modifications that may capitalize on chance [21]. Although it is possible to make a priori decisions to include some cross-loadings and correlated residuals when using ML [22]. ML estimation cannot account for all near-zero cross-loadings and near-zero correlated residuals that are often present in the measurement part of a model. A recent Bayesian approach proposed by Muthén and Asparouhov [20] is helpful in handling many of these concerns. In this approach, exact zeros are replaced with approximate zero informative priors to better reflect substantive theories. This Bayesian approach allows for the simultaneous estimation of all cross-loadings and residual correlations in an identifiable model, which is not possible with ML estimation [20].

For example, from a traditional perspective, maximum likelihood-structural equation modeling (ML-SEM) is applied to analyze the appropriate number of hidden indicators (constructs or latent variables) to determine the observed indicators. ML-SEM can facilitate concurrent analysis to illustrate the connection among observed indicators and the corresponding latent variables as well as the connections among latent variables (Ullman [23]). Disadvantages of ML-SEM, including the multivariate normal distribution of independent variables and small sample size, have encouraged researchers to seek better applications for prediction analysis.

Several studies suggest that Bayesian-structural equation modeling (B-SEM), which represents a nonparametric method, is able to overcome the limitations of ML-SEM [24, 25]. Lee and Song [26], Scheines, Hoijtink, and Boomsma [27], and Dunson [28] argued that Bayesian approaches assist with utilizing real prior information and information accessible in the observed data for enhanced results, better distribution of indices and statistics like percentiles and means of posterior distributions for unknown research parameters, and more trustable outputs for small samples.

Lee [29] suggested three advantages of the B-SEM approach: *a)* it leads to direct latent variable approximation, which is superior to traditional regression modeling for obtaining the factor score estimates, *b)* it models measurement indicators directly with their latent variables through familiar regression models, provides additional direct interpretation and allows the application of common techniques in regression modeling, such as outliers and residual analyses, and c) statistical modeling progress is based on the first moment properties of raw individual data, which are simpler than the second moment properties of the sample covariance matrix. Hence, B-SEM is easier to use in more complex situations.

In view of the above explanation, the main motivation of this study is to re-examine the UTAUT2 model in the context of e-learning via Facebook and test the data with stronger techniques for more accurate results. Considering the advantages and robust prediction power of B-SEM, UTAUT2 is examined in the context of e-learning and the results are compared with ML-SEM. The comparison offers additional knowledge about the predictive power of Bayesian techniques, consequently providing opportunities for future research. From another perspective, the current research results demonstrate the possibility to use Facebook for teaching and learning, whereby instructors can utilize it to connect, befriend and communicate with students, and extend the communicative activities of the traditional physical classroom into virtual forms.

The following section provides a related literature review with respect to prior research on e-learning, technology acceptance and a theoretical background of Bayesian analysis from an SEM perspective. Subsequently, the research method, data analysis and study results are specified. Based on the present research findings, the principal results are discussed together with the outputs prior to the final concluding remarks.

## Background of the study

A review of the literature indicates that research on technology acceptance and usage has been quite active, especially in recent years with social network/social media usage increasing very fast. Several models have been developed mainly in the information science domain to predict individual technology acceptance. Researchers have applied these models in a range of contexts. In the following section, previous literature related to e-learning and technology acceptance is discussed.

### Prior research on e-learning

Recently, e-learning has become a widely accepted learning approach [30]. This method of learning (E-learning) emphasizes the use of telecommunication technology for teaching and learning [31] and involves web-based communication systems. It enables learners to access various learning tools, such as discussion boards and document sharing systems anywhere, anytime [3]. E-learning comprises all forms of electronically supported learning and teaching processes [32]. In its broadest definition, e-learning includes instructions delivered via all electronic media, including the Internet, intranets, extranets, satellite broadcasts, audio/video, interactive TV, and CD-ROM [33]. Universities and educational organizations mostly use e-learning technologies to attain new and innovative ways of delivering education to students [4].

Social networks are one of the technologies that students use extensively to communicate with each other and share information. Social network sites are now adopted by many students as well as research scholars at academic institutions [14]. Social networks create the possibility for collaborative learning environments, which benefit learners and makes it easier, faster, more productive, and more memorable to meet, share and collaborate [34]. Sánchez, Cortijo, and Javed [9] indicated that Facebook is among the most popular SNSs among college students. Mason [35] stated that Facebook has many of the qualities desirable of an effective education technology (for teaching and learning) in its reflective element to use mechanisms for peer feedback and goodness-of-fit with the social aspects of university teaching and learning. It was found that educational use of Facebook is significantly related to its use for collaboration, communication, and resource or material sharing [12]. Student adoption and use of Facebook appear to be positively related with usefulness, ease of use, social influence, facilitating conditions and community identity.

Considering the fact that e-learning is related to telecommunication technology use for education and learning, several researchers of technology acceptance and education have conducted studies to identify the factors that affect students’ willingness to use such technology. Different theories/models have been applied to explain individual learning behavior in diverse contexts, such as Facebook [36], e-learning [37, 38], mobile learning [39, 40], iPad use for learning [41] and distance learning [42]. The following section discusses previous studies on technology acceptance.

### Prior research on technology acceptance

A number of researchers have employed the Unified Theory of Acceptance and Use of Technology (UTAUT) to explain factors that affect individual intention to use new technology. This theory was developed based on a comprehensive review of eight of the most common theories employed to predict computer use (Theory of Reasoned Action, Technology Acceptance Model (TAM), Theory of Planned Behavior (TPB), The Motivational Model, Combined TAM and TPB, Model of PC Utilization, The Innovation Diffusion Theory, and Social Cognitive Theory), based on conceptual and empirical similarities to predict individual adoption and use of technology [15]. UTAUT postulates that three core constructs, namely performance expectancy, effort expectancy, and social influence act as direct determinants of behavioral intention, while facilitating conditions and behavior intention are direct determinants of use behavior. It is argued that variables moderating these relationships are voluntariness of use, experience, age, and gender. In a study by Venkatesh, Thong, and Xu [16] three constructs were added to UTAUT, namely hedonic motivation, price value, and habit, and UTAUT2 was developed tailored to consumer IS adoption behavior. Venkatesh and Thong measured the determinants of intention to use technology in two stages. In UTAUT2, performance expectancy, effort expectancy, social influence, hedonic motivation and price value were considered predictors of individual intention, while facilitating conditions, habit and behavioral intention were considered determinants of technology use [16]. Venkatesh and colleagues defined performance expectancy as the degree to which individuals believe that using a system will help them attain gains in job performance, and they defined effort expectancy as the degree of ease associated with the use of new technology. Venkatesh and colleagues defined social influence as the degree to which individuals perceive that important others believe they should use the new system, while they argued that social influence is not significant in voluntary situations and only becomes significant when use is mandated by organizations. Based on UTAUT2, facilitating conditions are the degree to which individuals believe that appropriate organizational and technical infrastructure and facilities should exist to support new system use. They defined habit as the extent to which people tend to perform automatically because of learning, while hedonic motivation was defined as the fun or pleasure derived from using a technology [16]. Researchers have applied UTAUT and UTAUT2 in different contexts and settings to measure technology adoption and use behavior [43–45], while data was analyzed with an array of methods and techniques.

**Fig 1:**
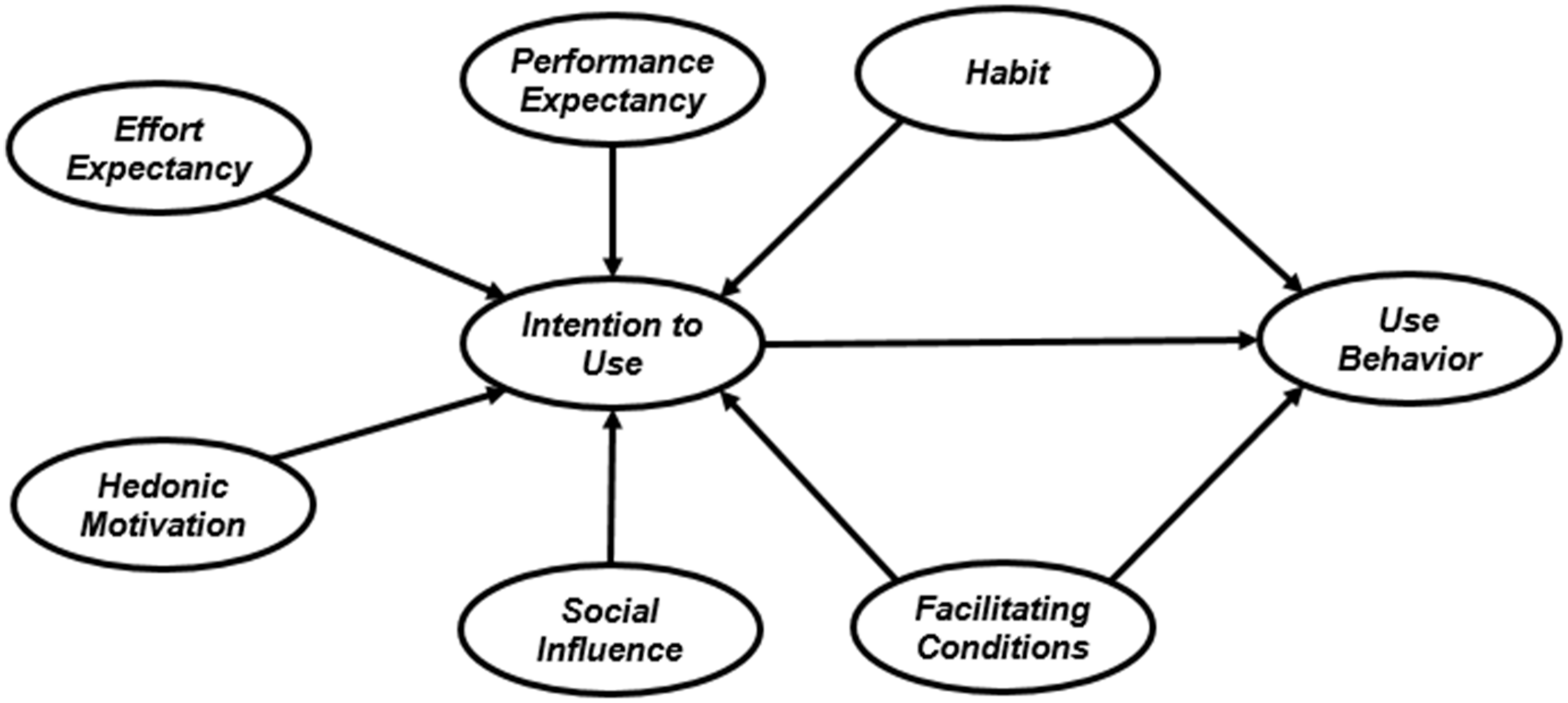
Research framework (UTAUT2; Source: Venkatesh, Thong, and Xu [16]).

In the current study, UTAUT2 (Fig 1) serves as a base model to examine students’ acceptance and use of e-learning via Facebook. UTAUT2 demonstrates the greater predictive power of Bayesian analysis compared to SEM in model testing.

The research framework includes eight constructs. Performance expectancy, effort expectancy, hedonic motivation, and social influence are the initial independent variables; use behavior is the main dependent variable; and intention to use acts as the mediator in both relationships between hedonic and facilitating conditions and use behavior.

### Theoretical Bayesian background

To clearly demonstrate how the Bayesian approach functions, besides the underlying model, a random sample ***Y*** = (*Y*_1_, …, *Y*_*n*_) is assumed from a distribution *f*(*y*|*θ*) with an unknown parameter, i.e. interest *θ* ∈ Ω. The primary goal from an estimation viewpoint is to estimate *θ* using the information available in sample ***Y***. To this end, the ML estimator can be used to estimate *θ* and obtain the ML estimate:

$${\hat{\theta }}^{ML}=\text{arg }{\text{min}}_{\theta }\;L\left(\theta \right)=\text{arg }{\text{min}}_{\theta }\;f\left(y_{1},\ldots ,y_{n}|\theta \right)$$ (1)

This classical means of estimating *θ* is influenced by the frequentist approach. Distribution models that rely on the frequentist approach for parameter estimation are classified as generative models [46]. These are employed to model the distribution of all available data assumed to have been generated with a fixed *θ*.

When parameter *θ* can be treated as a random variable, unlike with the frequentist estimation approach, in Bayesian analysis the researcher assigns a belief in the form of a probability statement to the parameter(s) of interest. It is worth noting that any statement about *θ* is made prior to data observation. Upon observing sample ***Y***, this probability statement about *θ* is updated by using the Bayes theorem. More specifically, a prior distribution *π* (*θ*) is assumed for *θ*. According to the chain rule, the joint probability of (***Y***, *θ*) is given by:

Joint distribution=Conditional disttribution×marginal distribution

To update the probability statement about *θ*, it suffices to evaluate *π*(*θ*|***y***), ***y*** = (*y*_1_, …, *y*_*n*_), which is the conditional distribution of *θ* given ***y***. Hence, according to the chain rule we have:

$$\pi \left(\theta |y\right)=\frac{f\left(y,\theta \right)}{f\left(y\right)}=\frac{f\left(y,\theta \right)}{\int f\left(y,\theta \right)d\theta }=\frac{f\left(y|\theta \right)\pi \left(\theta \right)}{\int f\left(y|\theta \right)\pi \left(\theta \right)d\theta }$$ (2)

The above is known as the Bayes theorem. The LHS of the Bayes theorem is called the posterior distribution of *θ*. Hence, the posterior distribution is proportional to the likelihood multiplied by the prior distribution as follows:

π(θ|y)∝L(θ)×π(θ) (3)

With Bayesian analysis, it is possible to assign probabilities to theories or models given the data, which is often the goal. With frequentist approaches, probabilities are assigned to the data given the theory or model, but they provide no information about the probability of the theory, model, or hypothesis [47].

$$\int \pi \left(\theta |y\right)\;d\theta =\int \frac{f\left(y|\theta \right)\pi \left(\theta \right)}{\int f\left(y|\theta \right)\pi \left(\theta \right)d\theta }d\theta =1$$ (4)

The above is a bona fide probability function, but this is not the case for the prior distribution. Since the prior distribution is a key part of any Bayesian analysis, focus remains on the various prior distributions in the forthcoming section.

#### Specifying the prior distribution

There are situations in which the researcher assigns a proper or improper belief to parameter *θ*. This belief can be collected from previous research, such as meta-analyses and studies with similar data, or it can reflect expert knowledge of researchers or practitioners [48]. Therefore, two categories of prior distributions are identified, namely subjective and objective. These consist of three main types of prior distributions that vary in their degree of (un)certainty about the parameter value of interest: non-informative, highly informative, and moderately informative [49]. When specifying highly informative priors, the researcher has a high degree of certainty about the parameter of interest and specifies numerical information about that parameter based on previous knowledge. These informative prior distributions are proper—that is, the function used as a prior density has a finite integral and is a probability density function (pdf). In contrast, non-informative priors are specified when the researcher has no prior knowledge about the parameter of interest and are improper—namely, the function used as a prior density has an infinite integral and is thus not a pdf. Finally, informative prior distributions are assigned to moderately informative priors, but scientific information about them is limited.

Well-known non-informative priors include Laplace’s prior, invariant prior, the Jeffreys prior, reference prior and matching priors. Among informative priors, conjugate priors are the most familiar. See Robert [50] for more details.

#### Bayesian computation

In this section, the well-known Markov Chain Monte Carlo (MCMC) numerical approach in Bayesian computation is briefly discussed. One elemental quantity that requires integration over a possibly high-dimensional parameter space is the Bayes theorem denominator. Thus, to generate a sample from the posterior distribution, the denominator needs to be solved. A practical computational tool for generating a sample from the posterior distribution is the MCMC simulation algorithm that uses π(θ|y)∝L(θ)×π(θ) to generate samples. The MCMC method constructs a Markov chain on state space *θ* ∈ Ω, whose steady-state distribution is the posterior distribution. It then returns a collection of *M* samples {*θ*^(1)^, *θ*^(2)^, …, *θ*^(*M*)^}, where each sample can be assumed to be drawn from *π*(*θ*|***y***). It is nonetheless important to note that MCMC is an iterative method, such that given the current state *θ*^(*i*)^, the algorithm makes a probabilistic update to *θ*^(*i*+1)^. There are a number of procedures in MCMC, with the two most general being the Metropolis-Hastings algorithm and the Gibbs sampler. Interested readers may refer to Marin and Robert [51] for more details.

Lee [29] extensively studied B-SEM, among others, and pointed out the advantages of B-SEM: a) a more flexible approach to deal with complex situations; b) utilizes useful prior information (if available); c) achieves reliable results with small/moderate sample sizes [26]; and d) gives direct estimates of latent variables. For further studies on B-SEM, please refer to Lee and Song [48] and Kaplan and Depaoli [52].

In this study, the research indicators collected are in the form of ordered categories. Yanuar, Ibrahim, and Jemain [53] suggested that before piloting a Bayesian examination, a threshold specification must be identified to treat the ordered categorical data as manifestations of a hidden continuous normal distribution. A brief explanation of the threshold specification is given below.

Suppose X and Y are defined as ***X*** = (***x***_**1**_, ***x***_**2**_, …, ***x***_***n***_) and ***Y*** = (***y***_**1**_, ***y***_**2**_, …, ***y***_***n***_), which can denote the ordered categorical data matrix and latent continuous variables, respectively. Moreover, the connection between ***X*** and ***Y*** is termed by applying the threshold specification. The procedure for *x*_1_ is described as an instant. More precisely, let

$$x_{1}=c\;if\;\tau_{c}-1<y_{1}<\tau_{c}$$ (5)

- *c* is the number of categories for ***x***_**1**_;
- *τ*_*c*_ − 1 and *τ*_*c*_ denote the threshold stages related to ***y***_**1**_.

For instance, in this work it is supposed that *c* = 3, which leads to *τ*_0_ = −∞ and *τ*_3_ = ∞. For the time being, the measures of *τ*_1_ and *τ*_2_ are evaluated based on the proportion of cases in each category of *x*_1_ using

$$\tau_{k}=\Phi^{-1}\left(\Sigma_{r=1}^{2}\frac{N_{r}}{N}\right),\;k=1,2,$$ (6)

In the current study, it is assumed that ***Y*** is distributed as a multivariate normal. In the above equation,

- ***Φ***^−**1**^(·) represents the inverse standardized normal distribution;
- *N* is the total number of cases;
- *N*_*r*_ is the number of cases in the *r*^th^ category.

Under B-SEM, $X=\left[\begin{array}{c} x_{1}\\ x_{2}\\ \vdots \\ x_{n} \end{array}\right]$ and $Y=\left[\begin{array}{c} y_{1}\\ y_{2}\\ \vdots \\ y_{n} \end{array}\right]$ are the ordered categorical data matrix and latent continuous variables, respectively, and $\Omega =\left[\begin{array}{c} \omega_{1}\\ \omega_{2}\\ \vdots \\ \omega_{n} \end{array}\right]$ is the matrix of latent variables. The observed data ***X*** are augmented with the latent data (***Y***, **Ω**) in the posterior analysis. The parameter space is denoted by $\;\Theta =\left[\begin{array}{c} \tau \\ \theta \\ \Omega \end{array}\right]$, where $\theta =\left[\begin{array}{c} \Phi \\ \Lambda \\ \Lambda_{\omega }\\ \Psi_{\delta }\\ \Psi_{\;\varepsilon } \end{array}\right]$ is the structural parameter. In line with Lee [29], the prior model is given by

π(Θ)=π(τ)π(θ)π(Ω|τ,θ) (7)

Where, due to the ordinal nature of thresholds, a diffuse prior can be adopted. For an approximately constant *c*, it is definite that:

π(τ)=c (8)

Moreover, from a subjective viewpoint, a natural conjugate prior can be implemented for *θ* with the conditional representation *π*(***θ***) = *π*(***Λ***│**Ψ**_**ε**_)*π*(***Ψ***_***ε***_). More specifically, let

$$\psi_{\varepsilon k}^{-1}∼\Gamma \left(\alpha_{0\varepsilon k},\beta_{0\varepsilon k}\right)$$ (9)

$$\left(\Lambda_{k}|\psi_{\varepsilon k}^{-1}\right)∼N\left(\Lambda_{0k},\psi_{\varepsilon k}H_{0yk}\right)$$ (10)

where *ψ*_*εk*_ is the *k*th diagonal component of *ψ*_*ε*_, *Λ*_*k*_ is the *k*th row of ***Λ***, and *Γ* denotes the gamma distribution. Finally, an inverse-Wishart distribution is adopted for *Φ* as follows:

$$\Phi^{-1}∼W_{q}\left(R_{0},\rho_{0}\right)$$ (11)

It is further supposed that all hyperparameters are known. The posterior distribution can be found by normalizing the product *L*(***Θ***│***X*** = *x*)*π*(***Θ***).

For sampling from the posterior distribution **Θ**|***X*** = *x*, MCMC is applied to deal with the computational complexity.

## Materials and method

With respect to the advantages and robust predictive power of the Bayesian approach in data analysis and for the purpose of measuring Facebook use for e-learning (re-examining the UTAUT2 model) among students, a questionnaire was developed. The data were collected via the questionnaire delivered to students who were taking a business statistics class at University of Malaya, Malaysia. The following sections explain the sampling procedure, measurement and an introduction to the Bayesian approach in more detail.

### Sampling and data collection procedures

The correspondents for the study include 170 bachelor students enrolled in a business statistics class at the Faculty of Business and Accountancy, University of Malaya, Malaysia.

To enable measurement of e-learning via Facebook, the class instructor created a business statistics Facebook group at the beginning of the semester. The lecturer provided the Facebook group address to the class, and all students requested to be part of this Facebook group within a week. This Facebook group is managed to facilitate e-learning material use by the students in the mentioned class. Every week, any information and supplemental materials related to the study subject are uploaded to the Facebook group, such as videos, texts, journal papers, and books. Students in this Facebook group ask their lecturer questions or communicate with classmates. The questionnaire was distributed at the end of the semester to 170 students who were using the Facebook group for learning in order to measure the students’ experience with using e-learning via Facebook. No information pertaining to the respondents’ names and identities was collected for this study, and the data were aggregated and analyzed anonymously.

### Measurements

In the current study, the original questionnaire developed by Venkatesh, Thong, and Xu [16] was applied and conceptualized to the e-learning context. The items related to the eight variables (S1 File) include use behavior, intention to use, facilitating conditions, habit, social influence, hedonic motivation, effort expectancy, and performance expectancy, which were adopted from Venkatesh, Thong, and Xu [16]. All indicators in this study were measured on a seven-point Likert scale (1 = strongly agree to 5 = strongly disagree).

## Results

The data analysis was based on the 170 questionnaires collected from students taking a business statistics class. The two approaches applied for data analysis are maximum likelihood and the Bayesian approach. The first modeling part was implemented using AMOS version 18, a flexible tool that allows examining the interrelationship under the normality assumption of the variables in the UTAUT2 framework for e-learning with Facebook. Second, B-SEM was employed with the same framework in the first part of data analysis along with WinBUGS (version 1.4) software. Four mathematical indices were applied to compare two outputs of the Bayesian and maximum likelihood estimators.

### Missing data, outliers, and normality

Missing data occurs when no value is stored for observation. Three data were missing from intention to use, habit, and effort expectancy. The missing data were replaced with the medians of the variables.

Outliers can be classified into two categories: simple and multivariate. Simple outliers have the highest values in connection with a single variable, whereas multivariate outliers only possess extreme values of a multiple variable on the surface. The Mahalanobis distance is an extremely general measure that is utilized for multivariate outlier measurement. If the Mahalanobis D-squared values, which can be calculated using AMOS or SPSS, are the highest, they tend to be the most probable significant outliers, meaning the outliers cause reduced analysis outcomes [54]. The significant outliers’ impact on the analysis needs to be assessed and investigated carefully to determine whether they can be retained. Byrne [55] suggestion for outlier analysis in SEM was taken in this study. Table 1 presents the Mahalanobis distance testing output. Case number 18 is the furthest from the centroid with a Mahalanobis D-squared value of 36.227. The p1 value indicates that, assuming normality, the probability of D-squared (for observation number 18) exceeding a value of 36.227 is < 0.0037. The p2 value, also assuming normality, reveals that the probability is still < 0.0091, which the largest D-squared value for any individual case would exceed 36.227. Given the wide gap in Mahalanobis D-squared values, the first five observations (numbers 18, 36, 48, 101, and 127) from other cases would be judged as outliers and deleted from further analysis. These outliers could affect the model fit, R^2^, and parameter estimates’ size and direction (see Table 1).

**Table 1:** Mahalanobis distance.

Observation number	MahalanobisD-squared	p1	p2
18	36.227	0.0037	0.0091
36	32.159	0.0124	0.0065
48	21.396	0.0268	0.0178
101	19.036	0.0714	0.0364
127	16.444	0.0934	0.0483
133	8.369	0.1236	0.0536
55	8.126	0.1297	0.0558

With SEM, the skewness and kurtosis indices are used for the normality test [56]. Byrne [57] mentioned that the absolute kurtosis values of skewness and kurtosis should be less than 2. Table 2 shows the kurtosis and skewness range indicators in their latent variables; the absolute kurtosis and skewness values are less than 2, therefore the indicators’ normality distribution is acceptable.

**Table 2:** Normality test of endogenous variables.

Variables	Skewness	Kurtosis
Performance Expectancy	[-1.36, 1.44]	[-0.96, 1.08]
Effort Expectancy	[-1.78, 1.68]	[-1.08, 1.43]
Facilitating Conditions	[-1.08, 1.22]	[-0.22, 1.03]
Hedonic Motivation	[-0.61, 1.12]	[-1.85, 0.71]
Social Influence	[-1.55, 0.73]	[-0.59, 0.81]
Intention to use	[-1.31, 1.09]	[-1.55, 1.07]
Habit	[-1.71, 1.49]	[-1.08, 0.31]

### Validity and reliability

Fornell and Larcker [58] determined the following terms and conditions for SEM validity and reliability:

1. Cronbach’s alpha-based validity. This index must be equal to or higher than 0.7 for every research model construct [59]
2. Reliability based on average variance extracted (AVE). This index must be equal to or higher than 0.50 for every research model construct [60].

Table 3 presents the Cronbach’s Alpha and AVE index values. Evidently, these values meet the recommended standards and norms. In conclusion, the research model validity and reliability are accepted.

**Table 3:** Validity and reliability based on AVE and Cronbach’s Alpha values.

Latent Variables	AVE	Cronbach’s Alpha
Performance Expectancy	0.616	0.789
Effort Expectancy	0.638	0.805
Facilitating Conditions	0.575	0.726
Hedonic Motivation	0.547	0.836
Social Influence	0.685	0.729
Intention to use	0.603	0.809
Habit	0.691	0.772

### Model fit

The data were run through Amos and the results (Table 4) indicate good data fit to the model. As seen in Table 4, the index outputs confirm that the measurement model significantly fits to the data.

**Table 4:** Goodness of fit analysis.

Index	Symbol/ or Abbreviation	Rules	Output
Normed Chi-square	[λ^2^/df]	[1, 3]	2.596
Comparative fit index	[CFI]	>0.90	0.925
Goodness of fit index	[GFI]	>0.90	0.901
Adjusted GFI	[AGFI]	>0.90	0.948
Incremental fit index	[IFI]	>0.90	0.911
Tucker Lewis index	[TLI]	>0.90	0.919
Root mean square error of approximation	[RMSEA]	<0.05 good fit <0.08 acceptable fit	0.043

**Fig 2:**
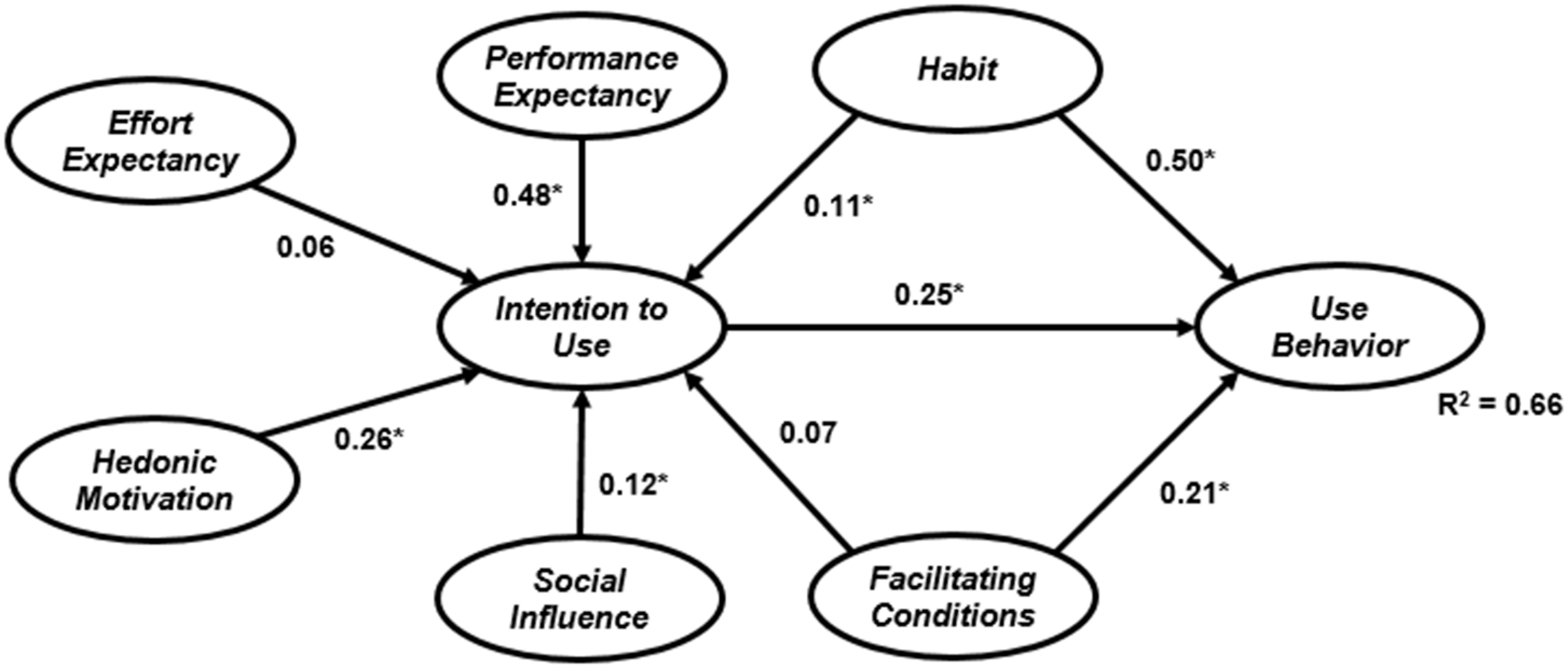
ML-SEM research model results(* represents the impact is significance [p-value < 0.05]).

**Fig 3:**
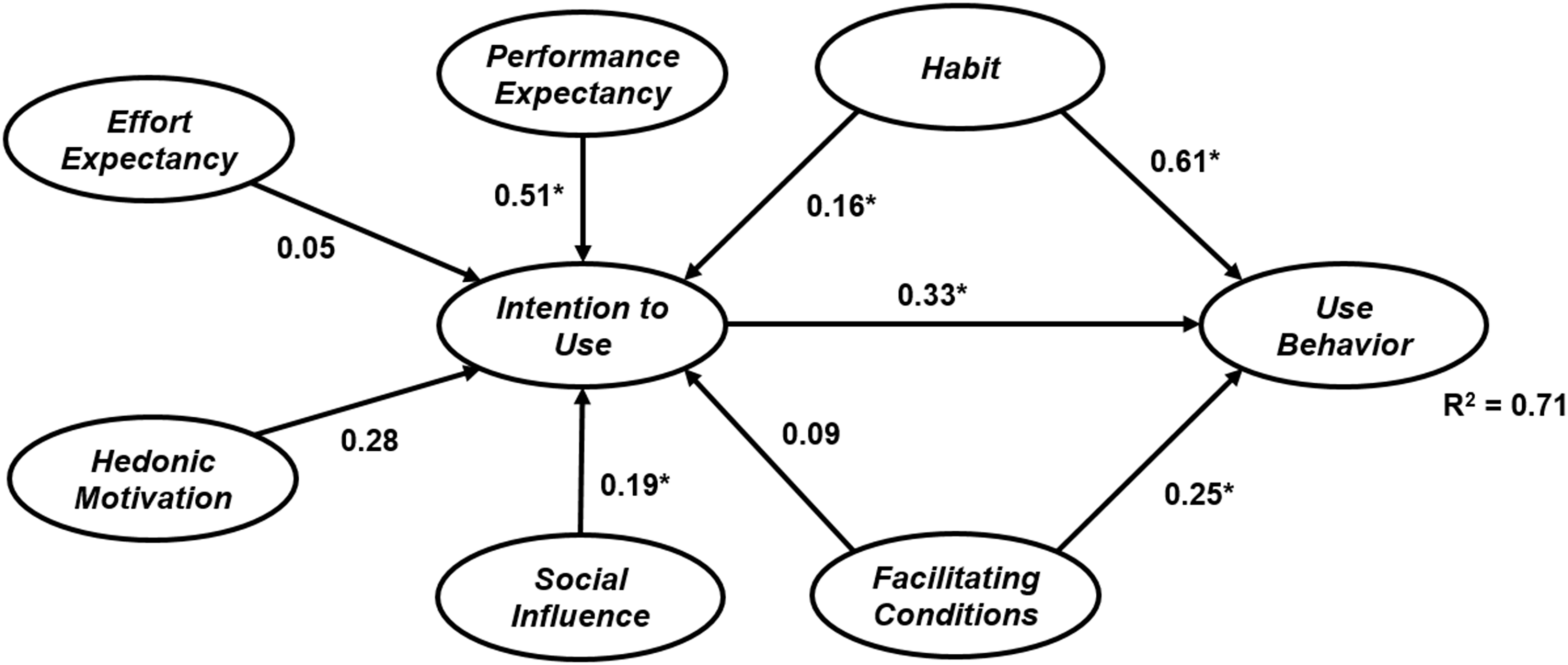
B-SEM research model results (* represents the impact is significance [p-value < 0.05]).

The data were run through both techniques and the results of both models are displayed in Figs 2 and 3. The figures show the estimated structural equations that address the relationships between latent variables for ML-SEM and B-SEM. According to the results, the relationship between performance expectancy, hedonic motivation, social influence, and habitual use, and intention to use e-learning via Facebook is significant in both models. Habitual use, facilitating conditions and intention to use have a significant positive relationship with e-learning via Facebook use. The effect of effort expectancy and facilitating conditions on intention to use is not significant for both models. A comparison of the two models signifies that B-SEM outperformed ML-SEM. ML-SEM was able to predict 66% of the variance in e-learning via Facebook use, while B-SEM predicted 71% of the variance in students’ e-learning via Facebook use. The beta value for all hypothesized relationships was stronger in the model tested with B-SEM. These results confirm the superior ability of B-SEM over the other technique.

In addition, the results indicate that performance expectancy is the strongest factor affecting students’ willingness to use Facebook for e-learning. In other words, students will use the new method if they think it will affect their academic performance or they will benefit from it. The habit of using Facebook is a strong predictor of use, which suggests that students use Facebook for different purposes, such as connecting with friends and family, socializing or learning. The effect of performance expectancy is not significant in both models, since young generations are familiar with technology and using Facebook is habitual, so they require no effort to use it. The significant effect of hedonic motivation demonstrates that it is important for students to have a pleasurable experience and enjoy the technology if they are going to use it. Social networks, especially Facebook, can satisfy this need and create an enjoyable experience for students. The non-significant effect of facilitating conditions on intention to use Facebook for e-learning indicates that the presence of facilities is more important for students if they are going to use it, and it does not influence their intention.

### Comparison between ML-SEM and B-SEM

This section presents a comparison analysis of the ML-SEM and B-SEM techniques in predicting the user behavior index in the UTAUT2 framework. Four indices were used to compare the two prediction techniques: root mean square error (RMSE), coefficient of determination (R^2^), mean absolute error (MSE) and mean absolute percentage error (MAPE). These are the most familiar statistical indices for modeling evaluation and are defined by the following equations:

1. Coefficient of Determination (R^2^)
  $$R^{2}=\frac{{\left[\sum_{i=1}^{n}\left(y_{i}^{,}-{\bar{y}}_{i}^{,}\right).\left(y_{i}-{\bar{y}}_{i}\right)\right]}^{2}}{\sum_{i=1}^{n}\left(y_{i}^{,}-{\bar{y}}_{i}^{,}\right).\sum_{i=1}^{n}\left(y_{i}-{\bar{y}}_{i}\right)}$$ (12)
2. Root mean square error (RMSE)
  $$RMSE=\;\sqrt[{2}]{\frac{\sum_{i=1}^{n}{\left(y_{i}^{,}-y_{i}\right)}^{2}}{n},}$$ (13)
3. Mean absolute error (MSE)
  $$MSE=\;\frac{\sum_{i=1}^{n}\left|y_{i}^{,}-y_{i}\right|}{n}$$ (14)
4. Mean absolute percentage error (MAPE)
  $$MAPE=\frac{1}{n}\sum_{i=1}^{n}\left|\frac{y_{i}^{,}-y_{i}}{y_{i}}\right|$$ (15)

In the above formulas, *y*_*i*_ is the *i*th actual value of the dependent variable and $y_{i}^{,}$ is the *i*th predicted value. Table 5 presents the values of the four performance indices, including R^2^, RMSE, MSE and MAPE for ML-SEM and B-SEM. The R^2^ value for B-SEM is greater than that for ML-SEM, and the RMSE, MSE and MAPE values for B-SEM are lower than those for ML-SEM. Therefore, the performance indices for the B-SEM technique indicate superior estimation to ML-SEM. The main reason B-SEM performed better is the neural network framework defined, which permits simultaneous self-adjustment of parameters and effective learning of the association between inputs and outputs in causal and complex models.

**Table 5:** ML-SEM and B-SEM comparison analysis.

	Performance Indices			
	R^2^	RMSE	MSE	MPE
ML-SEM	0.715	0.286	0.112	0.097
B-SEM	0.762	0.118	0.097	0.073

**Fig 4:**
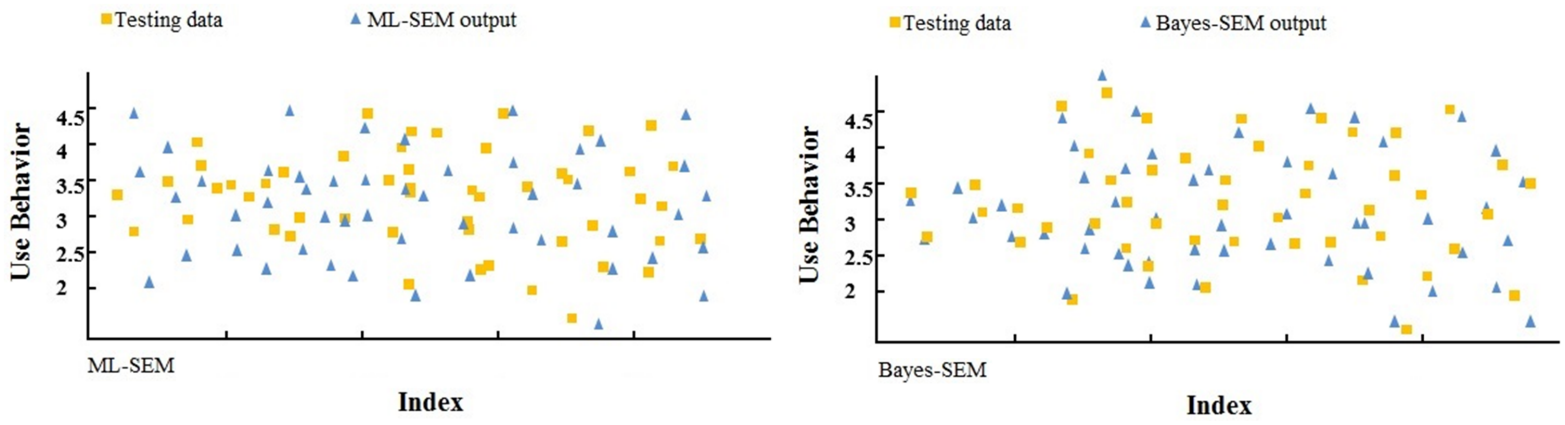
Scatter plots of predicted and measured values of use behavior in UTAUT2 [ML-SEM and B-SEM].

The scatter plots in Fig 4 illustrate that the B-SEM prediction values are closer to the real values than the ML-SEM predictions. The present comparative analysis proves that B-SEM has superior evaluation capability over ML-SEM in user behavior prediction under the UTAUT2 framework.

## Discussion

The main objective of this research was to demonstrate the power of the ML and Bayesian approaches with the SEM technique to predict students’ intention to use and Facebook use for e-learning based on the UTAUT2 model. These two approaches were compared in terms of prediction power and accuracy. ML-SEM was applied as a representative parametric modeling method, while Bayesian-SEM served as a representative nonparametric modeling technique to explore students’ use of Facebook for e-learning. Based on the UTAUT2 model, we measured the effect of performance expectancy, effort expectancy, hedonic motivation, social influence, facilitating conditions and habit on intention to use and Facebook use for e-learning. The results indicate significant effects of the determinants of intention to use and Facebook use for e-learning, but nonsignificant effects of effort expectancy and facilitating conditions. The significant effect of performance expectancy suggests that students would use Facebook for e-learning if they perceived a benefit from using it and that it would increase their academic performance or help them learn. The significant effect of hedonic motivation on intention to use highlights that if students enjoy e-learning via Facebook, it creates a pleasurable experience for them. The habit of using Facebook is an important factor for students, while using Facebook for e-learning is easy and effort expectancy is not an important factor for them. Habit is significant because students are used to Facebook and they normally utilize it for other purpose too, such as socializing with friends and communicating with others. It is also easy to use. Having appropriate facilities is important for students who wish to use Facebook for e-learning, but it does not influence their intention to use. This suggests that students need facilities, so help with, or providing facilities will affect use. This highlights the importance of providing e-learning materials over different channels for students to facilitate and encourage usage. The results of this study are consistent with the UTAUT2 findings.

Furthermore, re-examining the UTAUT2 model through the Bayesian and Maximum Likelihood approaches shows that the Bayesian approach produced better results in terms of the number of statistically significant parameters. The error statistics results also illustrate that the Bayesian-based framework with SEM provided a reasonably well-fitting model with a higher coefficient of determination (0.762) than ML-SEM (0.716). Compared with traditional methods, it was observed that introducing Bayesian statistics to traditional SEM improved model performance by reducing the RMSEA from 0.286 to 0.118, MSE from 0.112 to 0.097, and MPE from 0.097 to 0.073 (Table 5). Moreover, the values predicted based on B-SEM are closer to the actual values than ML-SEM (Fig 4). The results further demonstrate that the Bayesian framework model is less sensitive to sample size. The Bayesian model with SEM is a robust approach, since it does not require any distribution function assumptions such as normality. Therefore, this study suggests that a Bayesian approach can produce better results for testing UTAUT2 and predicting use behavior. In formulating ML-SEM and developing the Bayesian method, emphasis is on the raw individual random observations rather than on the sample covariance matrix. Moreover, Bayesian statistics is on the rise in mainstream psychology as well as management and information systems. It provides researchers with a number of theoretical and practical advantages over the “traditional” ML approach. As the Bayesian paradigm is further incorporated into information systems, researchers have access to methods uniquely suited to create cumulative knowledge.

## Conclusions and future research

This study was conducted to examine students’ use of Facebook for the purpose of e-learning. The data were tested with Bayesian analysis and compared to the ML approach. This study is unique from a methodological perspective, in that it is the first study to compare the ML estimator with the Bayesian estimator within an e-learning framework based on Facebook. This is a new research modeling contribution, given the increasing accuracy of alternative estimation and prediction techniques that employ software packages. The Bayesian approach allows researchers to model information system data meaningfully, and appears to offer greater statistical power than approaches that do not take censoring into account. In summary, this was the first study in which the flexible and innovative B-SEM approach was applied to evaluate various factor structures for predicting use behavior in UTAUT2 studies.

B-SEM can be applied to test hypotheses and theories and is capable of producing superior results to ML-SEM. This research also provides some directions to researchers who endeavor to apply B-SEM modeling. In this research, a practical method was used for structural and parametric learning. This methodology additionally provides guidelines for updating posterior probabilities with the generation of new evidence.

The results of the current study can benefit the academia, and teaching institutions and organizations in two ways. First, the presented results provide knowledge related to the effectiveness of social networks, especially Facebook, for teaching and learning. Academic managers and instructors can thus utilize social network sites and build platforms for effective communication between lecturers and students. In this respect, academic managers may use the findings of this research owing to the importance of providing these kinds of templates to enhance teaching quality in universities. The findings are also applicable to other organizations that need to train staff, and communicate or distribute information faster and more effectively. Second, this study created more knowledge related to data analysis and the higher predictive power of Bayesian analysis compared to maximum likelihood and regression, which can assist academics to obtain better results from their data.

In the current study, the lecturer distributed and collected the questionnaires, therefore any mistreatment of implementation reliability was minimal. However, the question of whether the findings can be generalized to other settings (subjects, times, places, etc.) is an important concern in any research. The structure of this research focused only on students, which may limit the ability to generalize the outputs of this study to other research settings like e-learning in organizations, institutes, and companies, since students are dissimilar from organizations and/or other individual users in some respects.

The notion of modeling the use behavior index in UTAUT2 studies by considering various indicators that describe the latent factors can be further explored by incorporating new survey data. This notion is particularly suitable with the sequential Bayesian approach if taking the results of this study into consideration as prior input for new surveys. Future studies can apply the findings from this study and use the B-SEM technique to analyze data, particularly in contexts that require stronger and more accurate results. In engineering for instance, neural networks and fuzzy sets are the most familiar techniques for non-parametric studies. These two methods and combinations of them may thus be suitable for future UTAUT2 studies.

## Supporting information

S1 Data Raw data. (XLS)

S1 File Research variables. (DOCX)
