# Supplementary material for: Testing students’ e-learning via Facebook through Bayesian structural equation modeling
Source: PLoS One. 2017 Sep 8;12(9):e0182311. doi: 10.1371/journal.pone.0182311 (PMC5590745; doi:10.1371/journal.pone.0182311)
Supplement: S1 File — (DOCX) [file pone.0182311.s002.docx]

| **Research Variables** |
| --- |
| **Performance Expectancy**  I find e-learning through Facebook to be useful in my study.  Using e-learning through Facebook enables me to accomplish tasks more quickly.  Using e-learning through Facebook increases my productivity.  Using e-learning through Facebook increases my chances of getting more positive learning benefit.  Using e-learning through Facebook gives me power of competitiveness.  **Effort Expectancy**  My interaction with e-learning through Facebook is easy to understand  It is easy for me to become skillful at using e-learning through Facebook.  Learning to operate e-learning through Facebook is easy for me.  I find e-learning through Facebook easy to use.  **Facilitating Conditions**  There are special supports (i.e. workshop) for using e-learning through Facebook for students in the university.  E-learning through Facebook is not compatible with other systems I use.  There is an external/internal support group available for assistance with e-learning through Facebook should I have any difficulties.  I have the knowledge necessary to use e-learning through Facebook.  I have resources necessary to use e-learning through Facebook in my study.  **Hedonic Motivation**  Using e-learning through Facebook is fun.  I have personal satisfaction in using e-learning through Facebook.  Using e-learning through Facebook is enjoyable.  Using e-learning through Facebook in my study is an attractive idea.  I am very enthusiastic to use e-learning through Facebook in my study.  Using e-learning through Facebook is very entertaining.  **Social Influence**  In general, the whole university has supported the use of e-learning through Facebook.  People who are important to me think that I should use e-learning through Facebook.  People whose opinions that I value prefer that I use e-learning through Facebook  People who influence my behavior think that I should use e-learning through Facebook.  **Intention to use**  I predict I will use e-learning through Facebook, if it is available in the future.  I will always try to use e-learning through Facebook in my study.  I plan to continue to use e-learning through Facebook frequently.  I plan to use e-learning through Facebook in my study in the next 3 weeks.  I intend to use similar e-learning through Facebook in the future.  I will learn to operate e-learning through Facebook in my study.  I will use e-learning through Facebook to achieve more opportunities in my life.  I will use e-learning through Facebook because I cherish the feeling of a useful service.  I will use e-learning through Facebook that enables me to finish my study successfully.  I have very seriously thought of using e-learning through Facebook in my study if it is available, within the next 2 months.  **Habit**  The use of e-learning through Facebook has become a habit for me.  I am addicted to using e-learning through Facebook.  I must use e-learning through Facebook all the time in my study.  Using e-learning through Facebook has become natural to me.  **Use behavior**  How many times per day do you post/update/share status on Facebook?  1) Not at all 2) 1 to 5 times  3) 6 to 10 times 4) 11 to 15 times  5) More than 15 times  On average, how much time per day do you spend on Facebook for learning purposes?  1) 1 hour or less 2) 1 to 2 hours  3) 2 to 3 hours 4) 3 to 4 hours  5) 4 hours and above  On average, ***how frequently*** do you normally use e-learning (any online material) through Facebook for the purpose of your study?  1) Not at all 2) less than once a week  3) about once a week 4) 2 or 3 times a week 5) 4 or 6 times a week 6) about once a day  7) more than once a day  On average, how much time do you spend to use e-learning material through Facebook in a day?  1) Not at all 2) 30 minute to 1 hour  3) 1 to 2 hours 4) 2 to 3 hours  5) 3 to 5 hours 6) more than 5 hours |
